# Responsiveness to 6-n-Propylthiouracil (PROP) Is Associated with Salivary Levels of Two Specific Basic Proline-Rich Proteins in Humans

**DOI:** 10.1371/journal.pone.0030962

**Published:** 2012-02-01

**Authors:** Tiziana Cabras, Melania Melis, Massimo Castagnola, Alessandra Padiglia, Beverly J. Tepper, Irene Messana, Iole Tomassini Barbarossa

**Affiliations:** 1 Department of Life and Environment Sciences, Macrosection of Biomedicine, University of Cagliari, Monserrato, Cagliari, Italy; 2 Department of Biomedical Sciences, University of Cagliari, Monserrato, Cagliari, Italy; 3 Institute of Biochemistry and Clinical Biochemistry, Catholic University, Rome, Italy; 4 Department of Food Science, School of Environmental and Biological Sciences, Rutgers University, New Brunswick, New Jersey, United States of America; German Institute for Human Nutrition, Germany

## Abstract

Thiourea tasting can be predictive of individual differences in bitter taste responses, general food preferences and eating behavior, and could be correlated with saliva chemical composition. We investigated the possible relationship between PROP bitter taste responsiveness and the salivary proteome in subjects genotyped for *TAS2R38* and gustin gene polymorphisms. Taste perception intensity evoked by PROP and NaCl solutions was measured in sixty-three volunteers (21 males, 42 females, age 25±3 y) to establish their PROP taster status, and 24 PROP super-tasters and 21 nontasters were selected to participate in the study. *TAS2R38* and gustin gene molecular analysis were performed using PCR techniques. Qualitative and quantitative determination of salivary proteins was performed by HPLC-ESI-MS before and after PROP taste stimulation. PROP super-tastings was strongly associated with the ‘taster’ variant (PAV haplotype) of *TAS2R38* and the A allele of rs2274333 polymorphism in the gustin gene and nontasting was associated with the minor alleles at both loci. ANOVA revealed that basal levels of II-2 and Ps-1 proteins, belonging to the basic proline-rich protein (bPRPs) family, were significantly higher in PROP super-taster than in nontaster un-stimulated saliva, and that PROP stimulation elicited a rapid increase in the levels of these same proteins only in PROP super-taster saliva. These data show for the first time that responsiveness to PROP is associated with salivary levels of II-2 peptide and Ps-1 protein, which are products of the *PRB1* gene. These findings suggest that *PRB1*, in addition to *TAS2R38* and gustin, could contribute to individual differences in thiourea sensitivity, and the expression of the PROP phenotype as a complex genetic trait.

## Introduction

Plants produce a large diversity of bitter-tasting compounds as protection against predation [1]. These substances include bitter alkaloids such as quinine and brucine, isothiocyanates from cabbage and mustard seeds, as well as certain fatty acids, amino acids and peptides, to name a few [2]–[4]. Since many bitter-tasting substances can be toxic, the ability of humans to detect bitterness at low concentrations represents an important evolutionary adaptation for limiting or avoiding the consumption plant foods that could be harmful [5]. On the other hand, several classes of bitter polyphenols found in tea, coffee, dark-colored fruit, citrus and chocolate [6] provide positive health benefits by acting as antibacterials and antioxidants [7].

Bitter taste is mediated by the TAS2R sub-family of G protein-coupled receptors [8], [9]. Humans posses ∼25 TAS2R bitter receptors encoded by clusters of genes located on chromosomes 5p, 7q, 12p [10]. So far, more than 550 ligands for human bitter receptors have been identified [11]. However, this number represents only a tiny fraction of the thousands of plant-based bitter compounds that exist in nature. Since the number of compounds greatly exceeds the number of receptors, it seems likely that individual receptors respond to more than one bitter compound type [12]. In fact, some receptors are narrowly-tuned, responding to a limited range of compounds. TAS2R8 is an example of a highly-selective receptor that has only 3 known ligands which share common structural properties. On the opposite end of the spectrum are TAS2R10, -14 and -46 which are highly promiscuous, responding to 50% of the bitter compounds applied in cell-based expression studies. TAS2R38, the receptor that binds the N-C = S moiety of the bitter thiourea compounds phenylthiocarbamide (PTC) and 6-n-propylthiouracil (PROP) [13], is considered modestly restrictive as this receptor also responds to compounds without the N-C = S motif [14].

Individual variation in the perception of bitter taste is a common human trait [6] that reflects the rich allelic diversity in TAS2R receptors. For example, sequence variation in *TAS2R19* has been associated with individual differences in the bitter taste of quinine [15]. Mutations in *TAS2R31* and *TAS2R43* (to a lesser extent) may be responsible for individual responses to the bitter aftertaste of saccharin and acesulfame-k [16], [17]. In addition, sequence variation in *TAS2R16*, *TAS2R19* and the haplo-block composed of *TAS2R3*, *-4*, *-5* are responsible for individual differences in the perception of alcohol, grapefruit juice and coffee, respectively [18].

Genetic variation in sensitivity to PTC and PROP, is the most- studied bitter-taste phenotype in humans [5], [19]. PROP responsiveness has been used as a general index of oral chemosensory perception since it associates with the perception of a wide range of oral stimuli including many of the bitter molecules discussed previously as well as, sweet substances, oral irritants and fatty texture [5]. PROP-related differences in chemosensory perception have been shown to influence food preferences which are the primary determinants of food selection and dietary behaviour [20]–[26]. Through this mechanism, PROP status is thought to play an important role in defining body composition and nutritional status [5]. Individuals can be defined tasters or nontasters based on their ability to discriminate threshold concentrations of PROP from plain water. When tested with suprathreshold (i.e., above threshold) concentrations of this compound, tasters can be further divided into those who are very sensitive, i.e. PROP super-tasters, and those who are moderately sensitive, i.e., medium tasters [27], [28].

The first molecular characterization of *TAS2R38* was accomplished by Kim et al. [13]. Three variant sites in this gene result in three amino acid substitutions (Pro49Ala, Ala262Val, and Val296Ile) and give rise to two common haplotypes: PAV, the dominant taster variant; and AVI, the nontaster recessive one. Individuals homozygous or heterozygous for the PAV haplotype taste PROP bitterness at low concentrations, whereas individuals who are either unable to taste PROP or who taste it only at high concentrations, are homozygous for the AVI haplotype. Other haplotypes (AAV, AAI, and PVI) that convey intermediate PROP/PTC response magnitudes have been rarely observed or limited to specific populations [29]. Since then, a growing number of studies have sought to fine-tune the genetic architecture of this phenotype [13], [29]–[32]. For example, studies have examined the effects of individual variant sites within the haplotype of the *TAS2R38* gene to better characterize their influence on bitter perception and to identify which sites may be critical for receptor activation [30], [33], [34].

Although the *TAS2R38* gene accounts for a large fraction of PROP/PTC phenotypic variation, it has become clear that other genetic loci contribute to the phenotype [15], [35], [36]. We recently showed that the polymorphism rs2274333 (A/G) of the gustin gene which controls the salivary protein carbonic anhydrase VI (CA6) alters the functionality of this enzyme and is strongly related to taste responsiveness to PROP [37]. In particular, allele A of this locus is strongly associated to the highest PROP responsiveness, whereas allele G is associated with the lowest one. Gustin is thought to be a taste-bud trophic factor and has long been implicated in taste function [38], [39]. In another study we showed how the combination of the *TAS2R38* and gustin gene genotypes modulate PROP phenotype, partially explaining supertasting [40].

Other salivary proteins have been implicated in bitter taste sensitivity. Fox [41] first suggested that the salivary composition might be responsible for individual differences in taste among people. On the basis of experiments showing that the stimulating capacity of a substance depends on its solubility [42], Fox hypothesized that taste blindness of nontasters may depend on the presence in their saliva of products (as proteins or colloids) which precipitate the taste substance and thus cause no taste to be perceived. It is known that salivary proline-rich proteins (PRPs) and histatins can bind and precipitate plant polyphenols in the oral cavity evoking astringency [43]–[45]. Genetic studies have shown that a cluster of *PRPs* genes, located at 12p13, are closely linked to a *T2R* gene cluster responsible for the ability to taste the bitterness of raffinose, quinine, cycloheximide, sucrose octaacetate and undecaacetate [46]–[49]. In addition, modification of the salivary proteome has been demonstrated in human responses to bitter tastants such as urea, quinine or calcium nitrate [50], [51]. At present, no studies have characterized the salivary proteome in individuals who vary in taste responsiveness to PROP. Given the importance of salivary proteins in taste function and the role that the PROP phenotype may play as a general marker of food selection and dietary behaviour, such studies are warranted.

The purpose of this work was to investigate the possible relationships between PROP taste responsiveness and the salivary proteome, before and after PROP bitter taste stimulation in individuals genotyped for *TAS2R38* and gustin gene polymorphisms.

## Results

**Figure 1:**
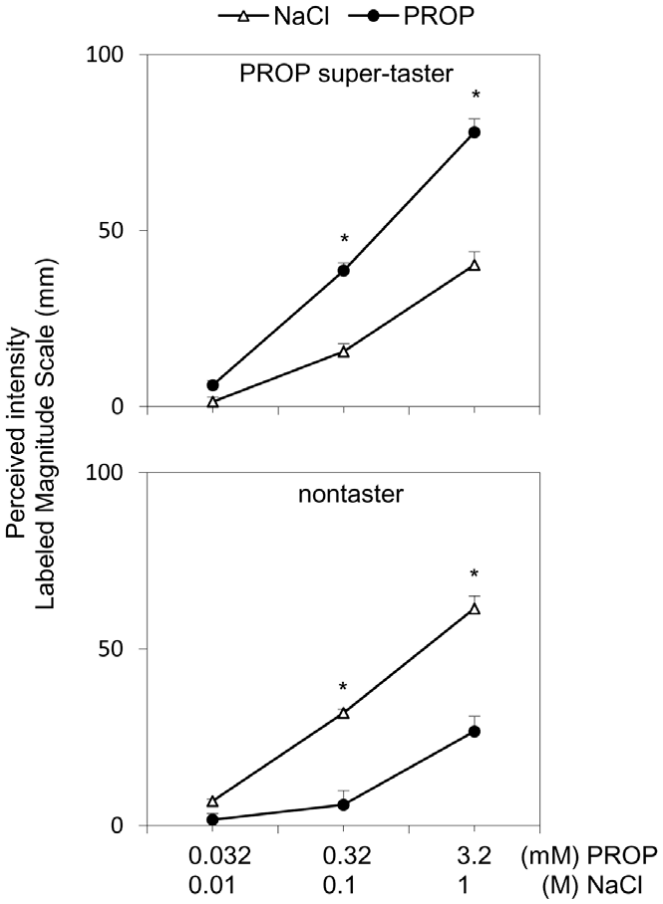
Classification of subjects by PROP taster status. All values are means (± SEM). Three-way ANOVA was used to compare PROP intensity ratings with NaCl intensity ratings in PROP super-tasters (*n* = 24) and nontasters (*n* = 21) (*p*<0.001). * indicates significant difference between PROP and the corresponding NaCl concentration (*p*<0.001; Newman-Keuls test). Medium tasters were not studied.

Figure 1 shows the PROP and NaCl intensity ratings of subjects classified as PROP super-tasters (n = 24) and nontasters (n = 21). ANOVA revealed a significant three-way interaction of Taster group×Solution type×Concentration on the intensity ratings (*F*_[2,258]_ = 37.89; *p*<0.001). Post-hoc comparisons confirmed that nontasters gave lower intensity ratings to the two highest PROP concentrations as compared to the two highest NaCl concentrations (*p*<0.001; Newman-Keuls test). Likewise, PROP super-tasters gave higher ratings to 0.32 and 3.2 mmol/l PROP as compared to the two highest NaCl concentrations (*p*<0.001; Newman-Keuls test).

Molecular analysis of the *TAS2R38* SNPs and the rs2274333 (A/G) gustin gene polymorphism showed that the two PROP taster groups differed statistically based on their allelic frequencies (*χ^2^* = 32.684; *p* = 7.999e-008; Fisher's test). In particular, PROP super-tasters had a very high frequency of haplotype PAV of *TAS2R38* (69%) and allele A of the gustin gene (93%), whereas nontasters had a higher frequency of haplotype AVI of *TAS2R38* (95%) and allele G of the gustin gene (60%).

**Figure 2:**
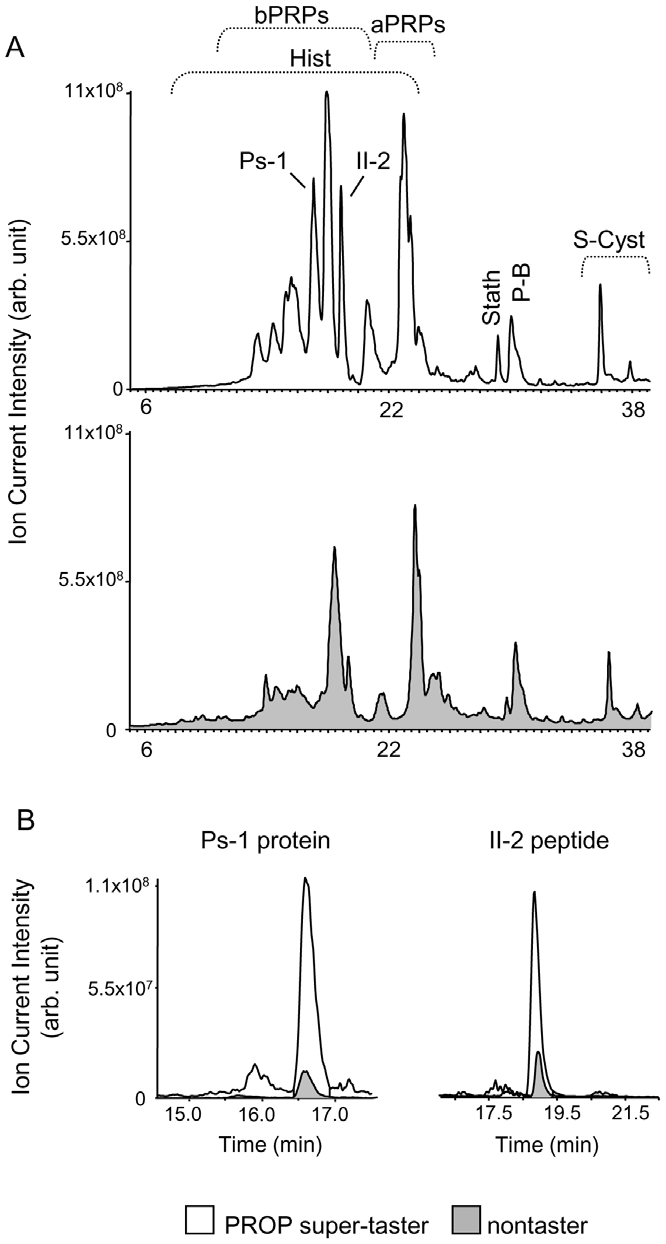
Examples of HPLC-MS profiles from un-stimulated saliva and extracted ion current peaks. (A) HPLC-MS Total Ion Current (TIC) profiles of the acidic-soluble fraction of saliva of a representative PROP super-taster (white profile) and nontaster (grey profile). (B) The ion current (XIC) peaks of Ps-1 protein and II-2 peptide extracted from the HPLC-MS profiles of the same subjects. The XIC peaks of the PROP super-taster (white filled) are superimposed on the same XIC peaks of the nontaster (grey filled).

HPLC-ESI-IT-MS analysis allowed us to demonstrate different relative concentrations of some proteins in the un-stimulated saliva of PROP super-taster subjects with respect to that of nontasters. An example of these differences is shown in Figure 2, where an HPLC profile (total ion current) of the acidic-soluble fraction of whole saliva of a representative PROP super-taster (white profile) and nontaster (grey profile) are shown in panel A. The extracted ion current (XIC) peaks of Ps-1 and II-2 proteins revealed in the two profiles are superimposed in Figure 2, panel B. The area of the Ps-1 protein peak corresponded to 3.2×10^9^ and 3.4×10^8^ arbitrary units, and the area of the II-2 protein peak corresponded to 1.8×10^9^ and 4.2×10^8^ arbitrary units in the PROP super-taster and nontaster saliva, respectively.

**Figure 3:**
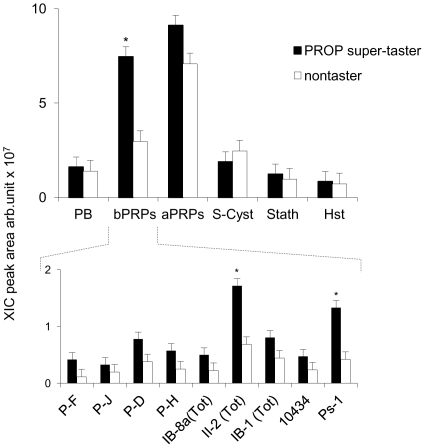
Relationships between PROP taste responsiveness and the basal level of salivary proteome. Mean values ± SEM of the XIC peak areas of the six protein families (P-B, bPRP, aPRP, S-Cyst, Stath, Hist) (upper graph), and of the following individual bPRPs (P-F, P-J, P-D, P-H, IB-8a Tot, II-2 Tot, IB-1 Tot, 10434 and Ps-1) (lower graph) in PROP super-taster (*n* = 24) and nontaster (*n* = 21) un-stimulated saliva. * = significant difference between PROP super-tasters and nontasters (*p*<0.001; Newman-Keuls test subsequent to two-way ANOVA).

Basal mean values ± SEM of the XIC peak areas of the six protein families (P-B, bPRP, aPRP, S-Cyst, Stath, Hist), as well as of the nine peptides of the bPRP family (P-F, P-J, P-D, P-H, IB-8a Tot, II-2 Tot, IB-1 Tot, 10434 and Ps-1) in un-stimulated PROP super-taster and nontaster saliva are shown in Figure 3. ANOVA revealed a significant two-way interaction of Taster group×Protein type on XIC peak areas of un-stimulated saliva proteins (the six protein families *F*_[5,258]_ = 5.80; *p*<0.001 and nine bPRPs *F*_[9,430]_ = 3.086; *p*<0.002). Post-hoc comparisons showed that, among the six protein families quantitatively determined, only the XIC peak area of bPRPs was significantly higher in PROP super-taster saliva than in nontaster saliva (*p*<0.001; Newman-Keuls test). Also, among the nine peptides of the bPRP family, only XIC peak areas of II-2 Tot and Ps-1were significantly higher in un-stimulated saliva of PROP super-tasters with respect to nontasters (*p*<0.001 and *p*<0.001, respectively; Newman-Keuls test). Importantly, the Ps-1 protein was entirely absent in 38% of nontasters. In addition, ANOVA revealed that the levels of all salivary proteins in un-stimulated saliva were not related to gender (the six protein families *F*_[5,258]_ = 0.98; *p* = 0.99 and nine bPRPs *F*_[9,430]_ = 0.30; *p* = 0.97).

**Figure 4:**
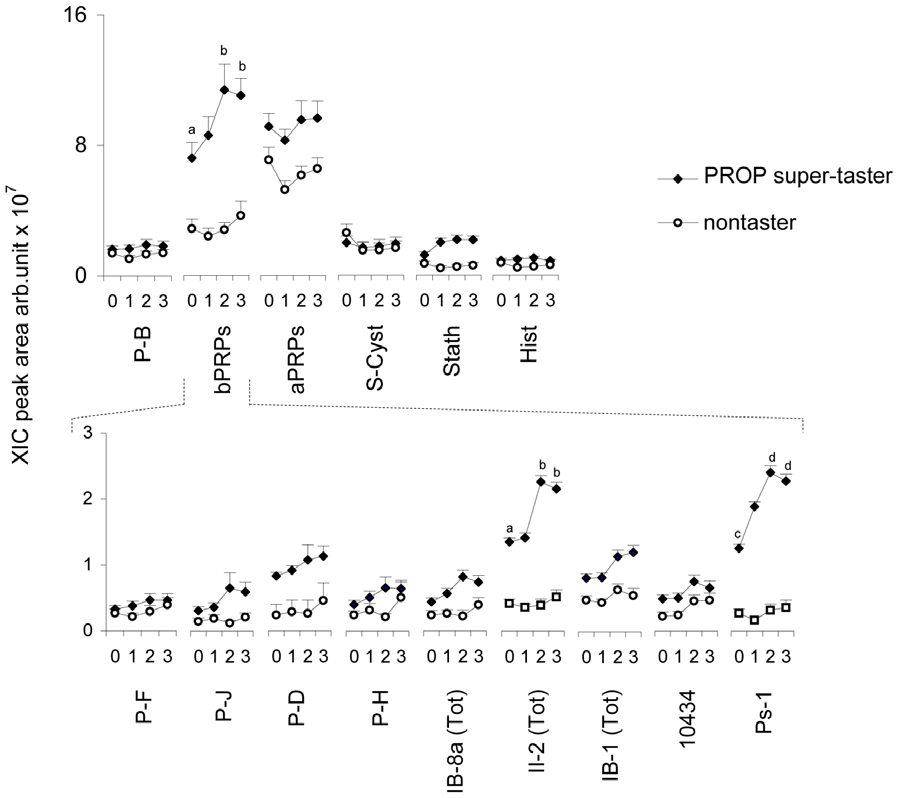
Relationships between PROP taste responsiveness and the salivary proteome after PROP bitter taste stimulation. Mean values ± SEM of the XIC peak areas of the six protein families (P-B, bPRP, aPRP, S-Cyst, Stath, Hist) (upper graph), and of the following individual bPRPs (P-F, P-J, P-D, P-H, IB-8a Tot, II-2 Tot, 10434 and Ps-1) in PROP super-taster (*n* = 24) and nontaster (*n* = 21) saliva before (0 in the X-axis) and after PROP (3.2 mM) stimulation. (The numbers 1, 2, 3 on the X-axis correspond to immediately after stimulation, after 5 and 10 min from stimulation, respectively). Different letters indicate significant difference (*p*≤0.025; Newman-Keuls test subsequent to three-way ANOVA).

Stimulated mean values ± SEM of the XIC peak areas of the six protein families (P-B, bPRP, aPRP, S-Cyst, Stath, Hist), as well as of the nine peptides of the bPRP family (P-F, P-J, P-D, P-H, IB-8a Tot, II-2 Tot, IB-1 Tot, 10434 and Ps-1) in PROP super-taster and nontaster saliva are shown in Figure 4. Post-hoc comparisons subsequent to three-way ANOVA showed that, among the six protein families quantified, taste stimulation with PROP (3.2 mM) induced, in PROP super-taster saliva, a significant increase in the XIC peak area of the bPRP family with respect to basal levels (after 5 min from stimulation, *p*<0.001, and after 10 min from stimulation, *p*<0.001 respectively; Newman-Keuls test). Among the nine peptides of the bPRP family, PROP stimulation induced a significant increase in the XIC peak area of II-2 (Tot) and Ps-1 proteins with respect to basal levels in PROP super-taster saliva (*p*≤0.025 and *p*≤0.0054 respectively; Newman-Keuls test). No significant changes were found in stimulated saliva of nontaster subjects (*p*>0.05).

## Discussion

A primary aim of the present study was to determine if the genetic predisposition to taste the bitterness of PROP is reflected in the salivary proteome. We demonstrated for the first time that PROP status was strongly associated with basal levels of specific salivary peptides belonging to the basic proline-rich protein family. In fact, a comparative analysis of salivary protein levels in un-stimulated saliva showed that PROP super-tasting, which is strongly associated with the PAV haplotype of *TAS2R38* and the A allele rs2274333 of the gustin gene, was also related to higher concentrations of the II-2 peptide and the Ps-1 protein, compared with PROP non-tasting which is associated with the minor alleles at both loci. None of the other proteins we analysed were related to PROP responsiveness, and no changes in the salivary proteome were related to gender. In addition, no changes in salivary protein secretion have been observed in the age range studied here [52]. Thus, neither gender nor age differences explain our findings.

The two bPRPs, that we found related to PROP status, are both encoded by the *PRB1* gene [53]. The family of *PRB* genes of chromosome 12p13.2 codes for basic and glycosylated PRPs [53], [54]. Mutations in *PRB* genes (including *PRB1*) are very common and could lead to lack of expression and null phenotypes. The *PRB1* gene shows different-length and null polymorphisms. In particular, this locus exhibits four alleles named *S, Small*; *M, Medium*; *L, Large*; and *VL*, *Very Large*. The alleles *S*, *M* and *L* have been characterized and their expression products are pro-proteins which generate mature bPRPs by post-translational proteolytic cleavages. It is known that II-2 peptide derives from the cleavage of each pro-protein expressed by *PRB1 S*, *M*, and *L* alleles. Conversely, Ps-1 protein only derives from the *PRB1 M* allele [53]. Our data on basal levels of Ps-1 protein in nontaster saliva indicate that this protein is poorly expressed (or not expressed) in these individuals, and suggest that the ability to taste PROP may be related to *PRB1* gene polymorphisms. In addition, these findings support the hypothesis that PROP super-tasting, which is related to high Ps-1 levels, might also be associated with the *M* allele of this gene. The latter assumption could also explain the specific increase in PROP super-taster saliva of the II-2 and the Ps-1 levels after PROP stimulation. By possessing a functional gene encoding the precursor for these proteins, PROP super-taster individuals may be able to secrete these proteins after stimulation, while nontasters lacking a functional gene are not able to do so.

In addition, since bPRPs are exclusively expressed by parotid glands [55], [56], our data suggest that the bitter taste of PROP may specifically stimulate the rapid salivary secretion of these glands. This is in agreement with previous data showing a taste-specific secretion of parotid glands following stimulation with sour-lemon [57].

Recently, we showed that PROP responsiveness is strongly associated with gustin (CA6) salivary protein functionality [37], and that the combination of *TAS2R38* and gustin gene genotypes partially explains supertasting [40]. The results of the present study confirm that PROP responsiveness is associated to *TAS2R38* and gustin gene polymorphisms [37], [40], and further extend this knowledge by examining salivary proteins which are products of the *PRB1* gene and are known to vary among individuals. These findings suggest that the *PRB1* gene may also play a role in modulating the expression of the PROP phenotype. Future studies will examine this possibility.

The salivary proteins, primarily PRPs, have been mainly studied in relation to ingestion of tannins [43], [58]–[61]. These salivary proteins neutralize the negative biological effects of tannins by favoring their precipitation [43]. Individuals who respond best to tannins are able to neutralize more of these compounds, as an adaptive mechanism. Having the ability to secrete high levels of these proteins would be a prerequisite to being a high-responder to tannins [59], [60]. Although the focus of this study is limited to a bitter molecule, such as PROP, our results show that PROP tasting could have implications in a broader nutritional context. Future studies should examine classically-defined bitter molecules as well as tannins. These studies will help to determine if these salivary proteins serve both a permissive function, that allows the individual to taste bitterness, as well as a protective function against the negative effects of tannins.

In conclusion, these novel findings extend the understanding of the PROP phenotype by identifying new candidates in the salivary proteome to explain individual differences in the genetic predisposition to taste thiourea compounds. Our finding may have important implications for understanding taste function impairment, eating behaviour and nutritional status. Whether the results described here are uniquely related to PROP tasting is unknown. Given the complex nature of human bitter taste experience, it seems likely that variation in the salivary proteome represents an additional layer of genetic diversity contributing to individual differences in bitterness perception. Future experiments will address this question by investigating other tastants and phenotypes.

## Materials and Methods

### Ethics statement

All subjects reviewed and signed an informed consent form. The study was approved by the Ethical Committee of the University Hospital of Cagliari, and has therefore been performed in accordance with the ethical standards laid down in the 1964 Declaration of Helsinki.

### Subjects

Sixty-three non-smoking volunteers (21 men and 42 women) were recruited through public advertisements at the local University. All were white, aged from 20 to 29 years and with body mass indices (BMIs) ranging from 18.6 to 25.3 kg/m^2^. Selected subjects had to have a stable weight (no variation of body weight larger than 5 kg over the previous 3 months). They were not following a prescribed diet or taking medications that might interfere with taste function. None of the subjects had food allergies, or scored high on eating behaviour scales (assessed by the Three-Factor Eating Questionnaire) [62]. In order to rule out any gustatory impairment, the threshold for the 4 basic tastes (sweet, sour, salty, bitter) was determined in all subjects. At the beginning of the protocol, each subject was verbally informed about the procedure and the aim of the study.

### PROP tasting

In order to classify each subject based on his/her PROP phenotype, PROP and sodium chloride (NaCl) ratings were collected using the 3-solution test [63], [64]. The test consists of three suprathreshold PROP (Sigma-Aldrich, Milan, Italy) (0.032, 0.32, and 3.2 mmol/l) and NaCl (Sigma-Aldrich, Milan, Italy) (0.01, 0.1, 1.0 mol/l) solutions dissolved in spring water. NaCl was used as a standard because taste intensity to NaCl does not change by PROP taster status in this method [63]. Solutions were prepared the day before each session and stored in the refrigerator until 1 h before testing.

### Molecular analysis

Subjects were genotyped for three single nucleotide polymorphisms (SNPs) at base pairs 145 (C/G), 785 (C/T), and 886 (G/A) of the *TAS2R38* that result in three amino acid substitutions (Pro49Ala, Ala262Val, and Val296Ile), and for the gustin (CA6) gene polymorphism rs2274333 (A/G) that consists of the substitution Ser90Gly. Molecular analyes were performed using PCR techniques followed by the sequencing of the fragments obtained in accord by Calò et al. [40]

### Salivary protein determination

#### Saliva treatment

Aqueous solution of trifluoroacetic acid (1 ml, 0.2%) was immediately added to 1 ml of each salivary sample in an ice bath in a 1∶1 v/v ratio, in order to preserve and stabilize the sample by inhibiting salivary proteases. The solution was then centrifuged at 8000 g, and 4°C for 15 min. The acidic supernatant was separated from the precipitate and either immediately analyzed by the HPLC-ESI-MS apparatus or stored at −80°C until the analysis. Sample size was 100 µL, corresponding to 50 µL of saliva.

#### HPLC-ESI-IT-MS analysis

The HPLC-ESI-MS apparatus was a Surveyor HPLC system (ThermoFisher, San Jose, CA, USA) connected by a T splitter to a photodiode array detector and the electrospray ionization/ion trap mass spectrometer LCQ Advantage (ThermoFisher, San Jose, CA, USA). The chromatographic column was a Vydac (Hesperia, CA, USA) C8 with 5 µm particle diameter (column dimensions 150×2.1 mm). The following solutions were utilized for RP-HPLC-ESI-MS analysis: (eluent A) 0.056% (v/v) aqueous TFA and (eluent B) 0.05% (v/v) TFA in acetonitrile-water 80/20, and the flow rate was 0.30 mL/min. Salivary proteins were eluted using a linear gradient from 0 to 54% of B in 39 min, and from 54% to 100% of B in 10 min. The T splitter permitted 0.20 mL/min to flow toward the diode array detector and 0.10 mL/min to flow toward the ESI source. The first five minutes of the RP-HPLC eluate was not transferred to the MS apparatus in order to avoid instrument damage derived from the high salt content. The photodiode array detector was set at 214 and 276 nm. Mass spectra were collected every 3 ms in the positive ion mode in the range 300–2000 m/z. The MS spray voltage was 5.0 kV, the capillary temperature was 260°C.

#### Identification of salivary peptides and proteins

Deconvolution of averaged ESI-MS spectra was automatically performed by using MagTran 1.0 software to obtain the experimental mass values [65]. These values were compared with the theoretical ones reported in the Swiss-Prot Data Bank (http://us.expasy.org/tools). Structural characterization of salivary proteins and peptides of interest, based on Tandem-MS analysis and automated amino acid sequencing of entire proteins, as well as of proteolytic fragments obtained after different enzymatic treatments of pure proteins, was performed as previously shown [66]–[70].

The six families of salivary proteins and peptides quantified in this study are listed in Table 1. We recently characterized a protein belonging to the basic proline-rich protein (bPRP) family with molecular weight of 23460 Da (unpublished results). Determination of its amino acid sequence confirmed that it corresponds to the Ps-1 protein previously described by Azen et al. [53].

**Table 1:** List of salivary proteins and peptides quantified by RP-HPLC-ESI-MS.

Name	Swiss-Prot code^a^	Experimental average mass (Da)
*Proline-rich peptide P-B*	(P02814)	5792.9±0.5
*Basic proline-rich protein family (bPRPs):*		
P-F	(P02812)	5843.0±0.5
P-J		5943.9±0.5
P-D	(P010163)	6949.5±0.7
P-H	(P02812/P04280)	5590.2±0.5
IB8-a (Tot):		
- IB8-a (Con1+)		11888±2
- IB8-a (Con1−)		11898±2
IB-1 (Tot):		
- IB-1	(P04281)	9593±1
- IB-1 nonphosphorylated		9513±1
- IB-1 Des-Arg_96_		9437±1
II-2 (Tot):		
- II-2	(P04280)	7609±1
- II-2 nonphosphorylated		7529±1
- II-2 Des-Arg_75_		7453±1
Protein with molecular weight of 10434 Da		10434±1
Ps-1		23460±3
*Acidic proline-rich phosphoprotein family (aPRPs):*		
PRP-1 type^b^ diphosphorylated	(P02810)	15515±2
PRP-1 type monophosphorylated		15435±2
PRP-1 type nonphosphorylated		15355±2
PRP-1 type triphosphorylated		15595±2
PRP-3 type^b^ diphosphorylated	(P02810)	11161±1
PRP-3 type monophosphorylated		11081±1
PRP-3 type nonphosphorylated		11001±1
PRP-3 type diphosphorylated Des-Arg_106_		11004±1
P-C peptide	(P02810)	4370.9±0.4
*Cystatin family (S-Cyst):*		
Cystatin S nonphosphorylated	(P01036)	14186±2
Cystatin S monophosphorylated (S1)		14266±2
Cystatin S diphosphorylated (S2)		14346±2
Cystatin SN	(P01037)	14312±2
Cystatin SA	(P09228)	14347±2
*Statherin family (Stath):*		
Statherin diphosphorylated	(P02808)	5380.0±0.5
Statherin monophosphorylated		5299.9±0.5
Statherin nonphosphorylated		5220.5±0.5
*Histatin family (Hist):*		
Histatin1	(P015515)	4928.2±0.5
Histatin1 nonphosphorylated		4848.2±0.5
Histatin 6	(P15516)	3192.4±0.3
Histatin 5	(P15516)	3036.5±0.3

#### Quantitative determination of salivary peptides and proteins

Salivary peptide and protein quantification was based on the area of the RP-HPLC-ESI-MS eXtracted ion current (XIC) peaks, measured when the signal/noise ratio was at least 5. The XIC analysis reveals the peak associated with the protein of interest by searching along the total ion current chromatographic profile, the specific multiply-charged ions generated at the source by the protein. The ions used to quantify the proteins/peptides were carefully selected to exclude values in common with other co-eluting proteins, and were the same as those reported in Cabras et al. [52]. The area of the ion current peak is proportional to concentration, and under constant analytical conditions can be used to quantify and compare levels of the same analyte in different samples [71], [72].

### Experimental procedure

The subjects were requested to abstain from eating, drinking and using oral care products or chewing gums for at least 8 h prior to testing that was carried out in three different visits. They had to be in the test room 15 min before the beginning of the session (at 9.30 AM) in order to adapt to the environmental conditions (23–24°C; 40–50% relative humidity) which were kept constant throughout the experimental session. In order to classify subjects for their PROP taster status, each subject was tested twice in different visits separated by a 1-month period. In women, testing was done on the sixth day of the menstrual cycle to avoid taste sensitivity changes due to the estrogen phase [73]. Stimuli were presented at room temperature as 10 ml samples. The order of taste stimulus presentation was reversed in the two visits. Samples within each solution type were tasted at random. Each stimulation was followed by oral rinsing with spring water. The interstimulus interval was set at 60 s. Taste intensity rating for each PROP or NaCl solution was collected using the Labeled Magnitude Scale (LMS) [74]. After tasting each sample, subjects placed a mark on the scale corresponding to his/her perception of the stimulus. The mean of the two replicates was calculated and the results were plotted for each subject. This procedure generates suprathreshold intensity functions for PROP and NaCl [63], [75]. When the PROP ratings increased more rapidly across concentrations than did the NaCl ratings, the subject was classified, as a “PROP super-taster”. Conversely, when the NaCl ratings increased more rapidly than did the PROP ratings, the subject was classified as a nontaster. When the PROP ratings overlapped with the NaCl ratings, subjects were classified as medium tasters. Medium tasters were excluded from participating in the proteome analysis in order to contrast the two extreme groups (PROP super-tasters and nontasters).

In the third visit, a sample (1 ml) of whole un-stimulated saliva was collected from each subject with a soft plastic aspirator as it flowed into the anterior floor of the mouth for less than 1 min, and then transferred to a plastic tube. One minute was sufficient to collect 1 ml of un-stimulated or stimulated saliva. Subjects then tasted 10 ml of PROP (3.2 mM). For complete impregnation of the oral cavity, subjects were instructed to keep the solution in the mouth for 5 s and then spit it out. After PROP taste stimulation, three samples of stimulated saliva were collected from each subject, immediately after stimulation, and at 5 and 10 min after stimulation.

### Statistical analyses

Three-way analysis of variance (ANOVA) was used to compare PROP intensity ratings with NaCl intensity ratings across PROP taster groups. The Newman-Keuls test was used for post-hoc comparisons.

Fisher's method (Genepop software version 4.0; http://kimura.univ-montp2fr/~rousset/Genepop.htm) [76] was used to test *TAS2R38* and gustin gene polymorphisms allele frequencies according to PROP status.

Two-way analysis of variance (ANOVA) was used to evaluate PROP super-tester nontaster differences in basal levels (un-stimulated saliva) of the six salivary protein families (P-B, bPRP, aPRP, S-Cyst, Stath, Hist), as well as of the following nine bPRPs: P-F, P-J, P-D, P-H, IB-8a (Tot), II-2 (Tot), IB-1 (Tot), 10434 and Ps-1. Two-way analysis of variance (ANOVA) was also used to evaluate gender differences in basal levels of the same six salivary protein families, as well as the nine bPRPs. The effects of PROP taste stimulation (immediately after stimulation, at 5 and 10 min after stimulation) on the levels of the same salivary proteins in PROP super-testers and nontasters were analyzed by three-way ANOVA. Post-hoc comparisons were conducted with the Newman-Keuls test. Statistical analyses were conducted using STATISTICA for WINDOWS (version 6.0; StatSoft Inc, Tulsa, OK, USA). *p* values <0.05 were considered significant.
